# Resolutions Adopted on the Death of Dr. Virgil O. Hardon

**Published:** 1904-04

**Authors:** Willis F. Westmoreland, Wm. Perrin Nicolson, John E. Gunn

**Affiliations:** Atlanta, Ga.; Atlanta, Ga.; Ex-officio; Atlanta, Ga.


### RESOLUTIONS ADOPTED ON THE DEATH OF DR. VIRGIL O. HARDON.

At a regular meeting of the Medical Board of "St. Joseph's Infirmary," held on Saturday evening, February 27th, a committee was appointed to draft suitable resolutions on the death of Dr. Virgil O. Hardon.

The following was the report:

WHEREAS, It has pleased God to call to himself Dr. Virgil O. Hardon, and to take from us a tried and trusted friend, a kind and skilful physician of warm-hearted and tender sympathies with all who suffered, a prudent and intelligent adviser and helper in everything tending to alleviate pain, and to widen and strengthen the usefulness of St. Joseph's Infirmary, and feeling deeply the loss of a man of such sterling qualities of mind and heart; therefore, be it

*Resolved*, by the Medical Board of St. Joseph's Infirmary—

1st. That in the death of Virgil O. Hardon this Infirmary has lost the man who did most for it in the days of its infancy and weakness; who clung to it most loyally in the period of its growth and development, and who guided it most wisely to its present completed organization and usefulness.

2d. That the Medical Board of this institution has been deprived of its esteemed, beloved and trusted President.

3d. That the Sisters in charge of St. Joseph's, and the members of the Medical Board, and the superintendent and class of nurses tender their sincere sympathy to the sorrowing wife of the deceased.

4th. That a copy of these resolutions be recorded on the minutes of the Medical Board, and also be given to the family of the deceased and to the medical and local press.

A committee of the Medical Board of St. Joseph's Infirmary.

WILLIS F. WESTMORELAND, M.D.,
WM. PERRIN NICOLSON, M.D.,
REV. JOHN E. GUNN, C.M., *Ex-officio*.

Atlanta, Ga., February 27, 1904.

